# 3′-UTR Sequence of Exosomal NANOGP8 DNA as an Extracellular Vesicle-Localization Signal

**DOI:** 10.3390/ijms25137294

**Published:** 2024-07-02

**Authors:** Manjusha Vaidya, Ayaka Kimura, Arjun Bajaj, Kiminobu Sugaya

**Affiliations:** Burnett School of Biomedical Sciences, College of Medicine, University of Central Florida, Orlando, FL 32816, USA; manjusha.vaidya@ucf.edu (M.V.); ay209975@ucf.edu (A.K.); arjun.bajaj@ucf.edu (A.B.)

**Keywords:** extracellular vesicles, genetic engineering, exosome-localization signal, NANOGP8 DNA, 22-base pair insertion

## Abstract

Extracellular vesicles (EVs) are garnering attention as a safe and efficient biomolecule delivery system. EVs intrinsically play a crucial role in intercellular communication and pathophysiology by transporting functionally active DNA molecules. The internalized DNA pleiotropically affects the recipient cells. Considering these salient features, an intentional incorporation of specific DNA gene cassettes into EVs and their subsequent delivery to the target cells has potential applications in genetic engineering. Moreover, efficient ways to insert the DNA into EVs during their biogenesis is valuable. Our current research is a step in the development of this technology. As such, cancer cells are known to secrete exosomes containing increased amounts of double-stranded DNA than normal cells. The clonal analysis in our previously published data revealed that exosomes released from various cancer cells contained a significantly larger population of NANOGP8 DNA with a 22-base pair insertion in the 3′-untranslated region (UTR) compared to those secreted by normal cells. This finding led us to hypothesize that the 22-base pair insertion may act as a signal to facilitate the incorporation of NANOGP8 DNA into the exosomes. To test this hypothesis, we compared the EV localization of an Enhanced Green Fluorescent Protein (EGFP) gene fused with the NANOGP8 3′-UTR, with and without the 22-base pair insertion. The quantitative PCR analysis showed a significantly higher EGFP DNA accumulation in exosomes released from cells transfected with the gene cassette containing the 3′-UTR with the 22-base pair insertion. The discovery of a DNA localization signal in exosomal DNA’s 3’-UTR could pave the way for the development of an EV-based DNA delivery system. This technology will open new possibilities in genetic engineering and innovative therapies using nucleic acid medicine.

## 1. Introduction

Cells secrete extracellular vesicles (EVs) to transport nucleic acids, proteins, lipids, and cell-signaling molecules for intercellular communication [1]. The EVs encompass subtypes such as microvesicles, apoptotic bodies, and exosomes, which share overlapping characteristics, although they may differ in size, biogenesis, secretion mechanism, and cargo [1,2]. The biomolecular contents of an EV reflect the state of its parent cell. For example, the EV-associated DNA reflects the pathological condition through altered quantities and differential sequences, thereby serving as a valuable biomarker for diagnosis, prognosis, and therapy response [3,4,5,6,7]. 

Regardless of the subtype, the bioactive molecules delivered by EVs, including DNA, exert a pleiotropic effect on the recipient. Our previous research indicates a reciprocal uptake of EVs in a co-culture of glioblastoma (GBM) and neural stem cells (NSCs). This internalization of EVs modifies the transcription of several stemness and drug-efflux genes in GBM and NSCs, underscoring the role of EV-delivered cargo in recipient cells [8]. 

EVs can be modified to augment the cargo-load-bearing capacity. Importantly, they can also be modified to enhance their specificity for the targets by incorporating organ-specific peptides onto their surfaces [9]. The EVs therefore serve as a biological delivery system, offering a significantly safer alternative to a viral delivery system [10]. EVs hold major advantages as a drug delivery system for therapeutics due to their innate stability, non-toxicity, bio-compatibility, and a low to nil immunogenic response evocation, combined with a high capacity for cell/tissue infiltration [11,12,13,14,15]. A highly desired characteristic of EVs for applications related to the central nervous system is their capability to traverse the intact blood–brain barrier. Garcia-Romero et al. have detected glioma-specific DNA marker sequences within the EVs obtained from the peripheral blood of patients [16,17]. Extensive research has proven that the EVs protect DNA from DNase-mediated degradation, making them an attractive delivery vehicle for genetic materials [18,19,20]. Consequently, researchers are exploring their potential for delivering therapeutic DNA for gene therapy in cancer and other diseases [21,22].

Proteins and RNAs are recognized as the most abundant biomolecules found in EVs, while DNA is comparatively scarce [23]. Elevated levels of DNA are detected in EVs under specific pathological conditions such as cancer, as compared to a normal state [24,25,26]. While the precise mechanism to localize DNA into an EV is not clearly understood, chromoanagenesis, the formation of micronuclei, may be associated with the encapsulation of cancer-specific aberrant DNA [27,28]. Although many researchers are exploring DNA delivery by EVs, the current method of introducing DNA into EVs involves either the endogenous overexpression of the DNA within the cell cytoplasm to generate sufficient molecules for a passive diffusion into EVs or exogenous introduction by transfecting the DNA into purified EVs [29,30]. Hence, the quest for analogous EV-sorting sequences identified in the 3′-untranslated region (UTR) of RNA molecules, facilitating their active transportation into EVs, is crucial for developing the technology to actively and effectively encapsulate DNA of interest within EVs [29]. 

**Figure 1:**
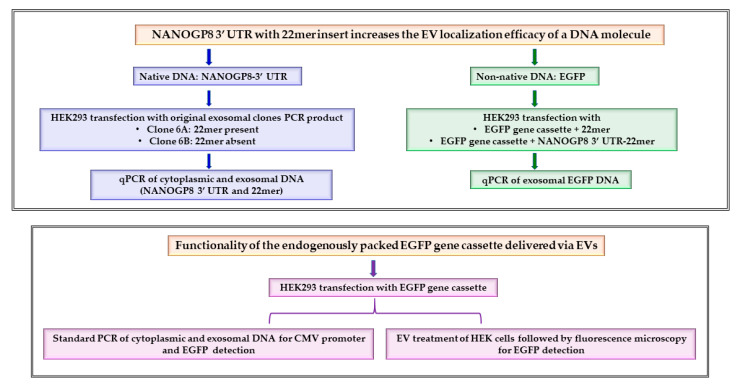
Outline of the research. Exosomal DNA 3′-UTR sequence as a DNA localization signal to extracellular vesicles.

Previously, we have reported that several cancer cells secreted a significantly larger population of the CD63-positive EVs, exosomes, containing DNA of NANOGP8, a pseudogene of the embryonic stem cell gene NANOG. The clonal sequencing analysis revealed that the exosomal NANOGP8 DNA contained a peculiar 22-base pair insertion (22mer) in its 3′-UTR [4]. Since cancer cell-derived EVs are reported to contain more DNA than the ones produced by non-cancer cells, we hypothesized that the 22mer in the 3′- UTR could be a localization signal transferring the NANOGP8 DNA to EVs [24]. The 3′- UTR plays a crucial role in mRNA subcellular localization, including localization to EVs [25,26,31,32,33]. However, the involvement of a specific sequence or 3′-UTR of a gene in DNA localization to EVs or exosomes remains unexplored. In the current research, we have fused the 22mer-containing NANOGP8 3′-UTR to a CMV promoter-driven EGFP gene cassette to confirm the efficacy of the EGFP DNA localization into EVs/exosomes and to test the functionality of the EV-delivered gene cassette in the target cells. If our hypothesis is validated, this novel localization sequence could provide unique advantages for delivering DNA more efficiently into the cells by EVs/exosomes. Figure 1 illustrates the steps involved in our research.

## 2. Results

Throughout this paper, the terms EVs and exosomes are used to denote the cell-secreted nano-vesicles. When the EVs are further immunopurified using a CD63 antibody, they are denoted as exosomes. The results remain consistent regardless of the terminology used. Exosomes/EVs are not known to have a homeostasis mechanism to regulate the DNA population. For example, as stated earlier, the cancer cells secrete EVs with elevated amounts of DNA compared to the normal cells. Consequently, the EVs also lack a stable expression of housekeeping genes or any other type of DNA that could be available for the reference and normalization of the DNA cargo. Due to the lack of an endogenous control during thermocycling, ∆Ct, which normally refers to the difference between the target gene and the reference gene, cannot be calculated for the DNA samples in EVs. Therefore, all the qPCRs performed in the study are normalized to the protein content of the EV samples before the amplification reactions and the cycle threshold (Ct) values are presented. 

### 2.1. The 22mer Insert Does Not Affect the Localization Efficacy of the NANOGP8 3′-UTR DNA to Exosomes

**Figure 2:**
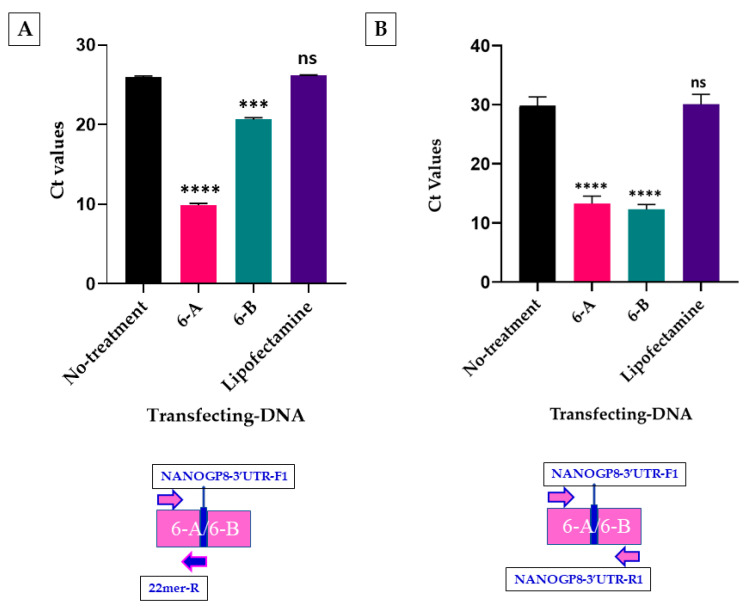
Graph depicting the Ct values of exosomal NANOGP8 3′-UTR DNA amplified via qPCR. Exosome samples used in the qPCR were normalized to their protein content. Ordinary One-way ANOVA was performed for multiple comparisons of other types of DNA to the values of the non-treated sample, followed by a post hoc Dunnett’s test to compare the mean of 6A non-treated sample’s Ct values to the rest of the DNA samples. (**A**) NANOGP8 3′-UTR forward and 22mer reverse primers: (Adjusted *p* value: ****: *p* < 0.0001, ***: *p* = 0.0004, no-significance (ns) = 0.565). (**B**) Both the primers used in the qPCR amplification were also used in creating the original 6A/6B clones. NANOGP8 3′-UTR forward and reverse primers: (Adjusted *p* value: ****: *p* < 0.0001, ns: *p* = 0.7726).

The qPCR of NANOGP8 3′-UTR DNA in exosomes upon transfection of the HEK293 cells with the PCR products of 6A (22mer present) and 6B (22mer absent) revealed that 6B has an enhancement effect on the exosomal localization of endogenous 3′-UTR DNA. Because 6B does not contain the 22mer, and the 22mer itself was used as one of the primers; the Ct value of the exosomal DNA amplification for 6B should have been close to the Lipofectamine or the no-treatment samples, and not significantly lower as the graph shows (Figure 2A). However, there is no significant difference in the Ct values of exosomal 6A and 6B DNA after transfection when another NANOGP8 3′-UTR primer pair flanking the 22mer insert is employed (Figure 2B). With these primers, the Ct values of 6A and 6B are remarkably lower than that of the non-treated or the Lipofectamine-treated samples. This result shows that the 22mer present in the NANOGP8 3′-UTR DNA does not influence the localization of the flanking sequences of native DNA.

### 2.2. The EV-Localization of NANOGP8 3′-UTR-End Containing 22mer Is Independent of Its Cytosolic Concentration

**Figure 3:**
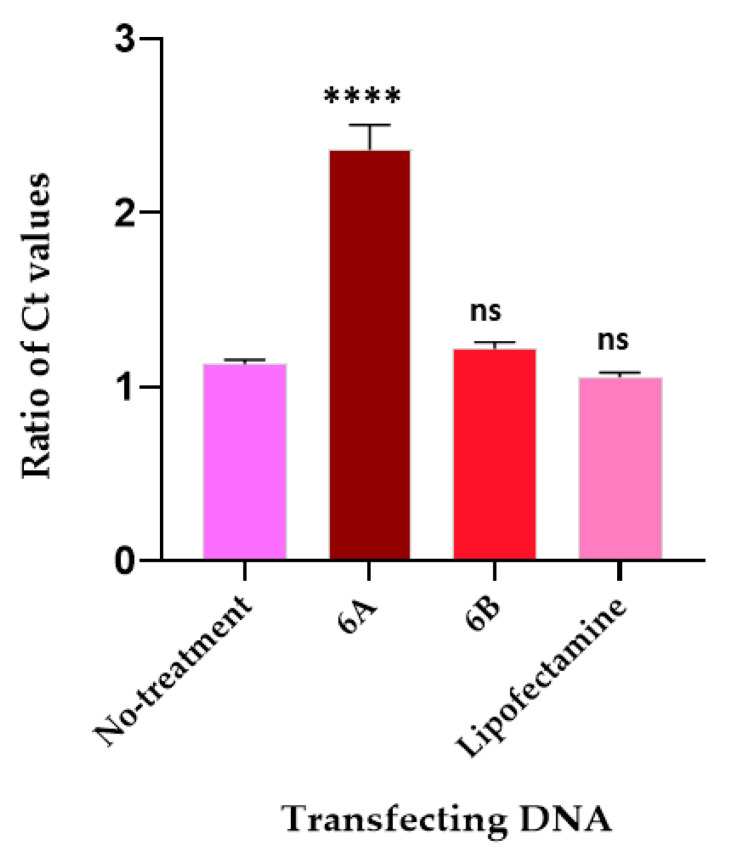
A graphical depiction of the ratio between the Ct values of EVs and the cytoplasmic 22mer DNA. Ordinary One-way ANOVA was performed for multiple comparisons of the ratio of non-treated sample to 6A, 6B, and the Lipofectamine treated samples. A post hoc Dunnett’s test was used to compare the mean of the non-treated sample ratio to the ratios of the rest of the samples. No treatment vs 6A: Adjusted *p* value: ****: *p* < 0.0001, no treatment vs 6B: Adjusted *p* value: ns: *p* = 0.1411, and no treatment vs Lipofectamine: Adjusted *p* value: ns: *p* = 0.2491. (ns = non-significant).

We performed a qPCR analysis to compare the quantities of 22mer in the cytoplasm and the EVs after transfection with 6A and 6B DNA. The primers described in Figure 2A are employed in the amplification. The ratios of Ct values of EV-22mer to the cytoplasmic 22mer are depicted in the graph in Figure 3. Upon transfection with the NANOGP8 3′-UTR alone (6B), the EV–cytoplasm Ct value ratio for the 22mer is close to the values for Lipofectamine and the non-treated samples (~1.0). The physiological concentrations of the UTR DNA in the cytoplasm and its localization to EVs remain the same after the addition of 6B. However, the addition of the UTR DNA containing 22mer (6A) increases the value of Ct ratio to 2.4. Increased concentrations of 22mer-containing UTR in the cytoplasm do not result in elevated UTR amounts in EVs even though the 22mer is present.

### 2.3. EGFP Gene Cassette Endogenously Packed in EVs Is Functional in the Recipient Cell

The sanger sequencing performed using GENEWIZ^®^ from Azenta Life Sciences and the BLAST analysis of the PCR product obtained using the template of the EGFP plasmid confirmed that the amplified region belonged to the EGFP gene (Figure S1). The translational functionality of the EGFP cassette under the CMV promoter was tested before attaching the PCR products of 6A and 6B to it. 

**Figure 4:**
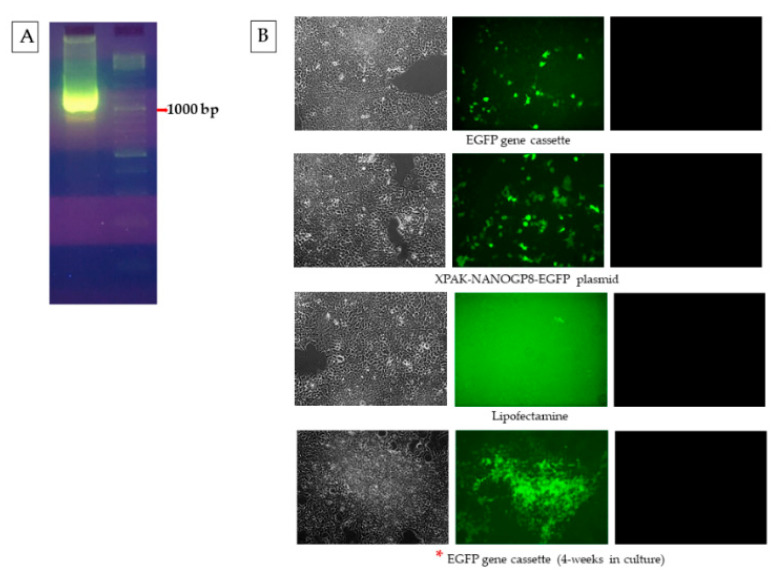
The HEK293 cells transfected with EGFP gene cassette express the fluorescent protein. (**A**) Gel electrophoresis: PCR product of CMV promoter-EGFP gene cassette amplified using Vector Builder’s pRP[Exp]-EGFP/Neo-CMV>{Tag/hNANOG[NM_024865.2]* plasmid (referred to as XPAK-NANOGP8-EGFP) (vector ID #VB170211-1008gnb) as template. The forward primer is located 47 base pairs upstream of the promoter, in SV40 late pA. The reverse primer includes only the EGFP gene, without the end codon. The PCR product was eluted from the gel and the DNA was used for transfection. The same PCR product was also cloned into the sequencing vector, pCR4-TOPO-TA, to confirm the presence of CMV and EGFP sequences. (**B**) Images of HEK cells 24 h after transfection with EGFP gene cassette (10× magnification). The last row labelled with a red asterisk shows the HEK293 cells that continued to express the protein after 4 weeks. The EGFP expression was detected at the Excitation 488 nm/Emission 509 nm. To confirm the fluorescence is from the EGFP and not an artifact, the cells were also exposed to the RFP laser line with Excitation 532 nm/Emission 588 nm.

The HEK293 cells, upon transfection with the EGFP gene cassette, showed green fluorescence when imaged with fluorescence microscopy, confirming that the cassette expresses the protein and that there is no loss of function during the process of its creation (Figure 4). The cells transfected with the original plasmid XPAK-NANOGP8-EGFP, used in making the cassette, also expressed the EGFP, confirming that the process of transfection worked well. The HEK293 cells continue to express the protein after 4 weeks in the culture.

**Figure 5:**
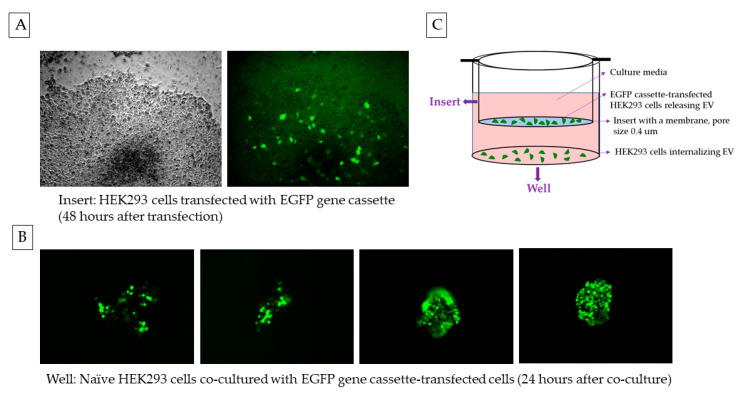
EGFP gene cassette endogenously packed in EVs is functional in the recipient cell. (**A**) HEK293 cells transfected with EGFP gene cassette, 48 h after transfection expressed the protein. To avoid any premature exposure of the naïve cells to the EGFP cassette DNA, the cells in the well were transfected separately before the co-culture began. (**B**) The naïve (recipient) cells in the well expressed the florescent protein upon co-culture with the transfected cells in the insert. (**C**) During the co-culture, the transfected cells and the naïve cells were separated from each other by a cell membrane with a pore size of 0.4 µm. The EGFP expression was detected at the Excitation 488 nm /Emission 509 nm. The cells were imaged under 10× magnification.

When the EGFP expressing HEK293 cells (cultured in cell insert) were co-cultured with the naïve HEK293 cells (cultured in well), the latter showed a green fluorescence in the fluorescent imaging, suggesting that the target cells received the RGFP expression cassette DNA secreted by the transfected cells. The DNA packed in the EVs may have been transcribed after internalization of the EVs (Figure 5). Our previously published research has described the uptake of the EVs in a co-culture and their subsequent internalization affecting the recipient [8].

### 2.4. CMV-EGFP DNA Is Present in the Exosomes of HEK293 Cells after Transfection

**Figure 6:**
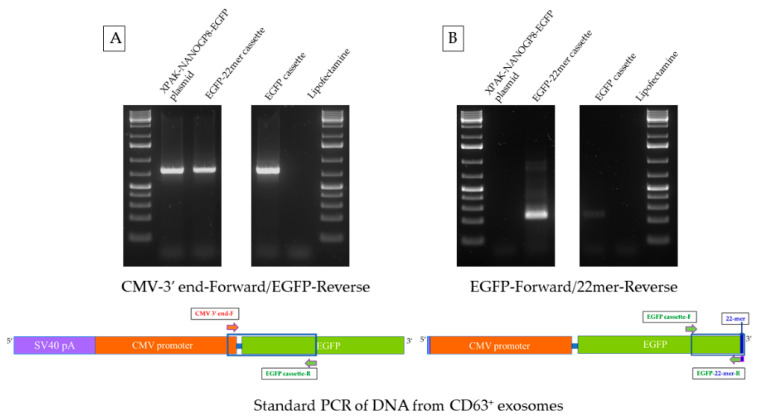
Standard PCR of CD63^+^ exosomal DNA using CMV promoter, EGFP gene, and 22mer primers to detect the DNA qualitatively. (**A**) The forward primer is located at the 3′- end of the CMV promoter and the reverse primer is located in the EGFP gene. (**B**) The forward primer is located in the EGFP gene whereas the reverse primer is made with the 22mer sequence and a few nucleotides from the EGFP gene. The blue boxes indicate the part of the cassette amplified.

The CD63^+^ exosomes of HEK293 cells, after transfection with the gene cassette, pack the DNA for CMV promoter and the downstream EGFP gene, with or without 22mer attached. A standard PCR test of exosomal DNA, performed using a forward primer at the CMV-3′ end and a reverse primer in the EGFP gene, brought out a PCR product from all types of exosomes, i.e., the cells transfected with the EGFP cassette, EGFP-22mer cassette, as well as the XPAK-NANOGP8-EGFP plasmid, had the presence of this DNA (Figure 6A). The same exosomal DNA samples, amplified with a forward primer located in the EGFP and the reverse primer made of the 22mer sequence, showed a PCR product only in the exosomes of HEK293 cells transfected with EGFP-22mer, but not the plasmid or the EGFP cassette, as expected. The plasmid and the original EGFP cassette both lack the 22mer (Figure 6B).

### 2.5. Naïve HEK293 Cells Express EGFP upon Treatment with Exosomes Containing CMV-EGFP DNA

**Figure 7:**
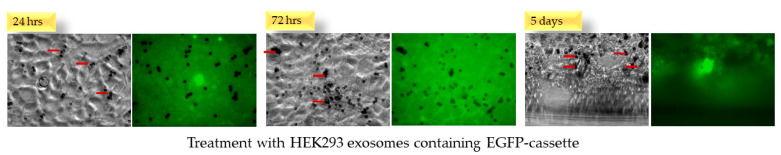
Fluorescent microscopy images of the HEK293 cells treated with exosomes containing the EGFP cassette. The red arrows denote the CD63 antibody conjugated magnetic beads. The exosomes captured using theses beads are used in the treatment of the naïve cells. (Excitation 488 nm/Emission 509 nm). The cells are imaged under 40× magnification.

The HEK293 cells, when treated with exosomes containing EGFP cassette, showed a dull fluorescence during the first 3 days. However, the fluorescence signal improved after 5 days of continued incubation (Figure 7). 

### 2.6. Mer Insert Sequence Alone Is Not Enough for an Effective EV-Localization of A DNA

Because more of the 22mer insert was found in NANOGP8 3′-DNA in cancer cell-secreted exosomes, we wanted to check the exosome-localization capability of the insert alone. Upon transfection with the EGFP-22mer cassette, a standard PCR amplification of the cytoplasmic DNA of co-cultured HEK cells using the CMV promoter and EGFP-specific primers confirmed the presence of the gene cassette in both the transfected cells in the insert (I), as well as the treated (originally naïve) cells in the well (W) (Figure S2). The naïve cells likely received the cassette via EVs secreted by the transfected cells. 

**Figure 8:**
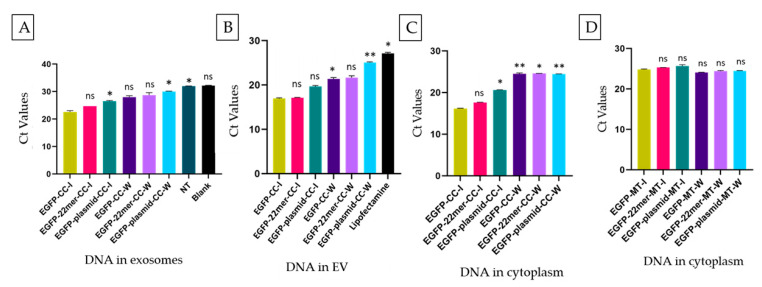
A qPCR of HEK293 exosomal, EV, and cytoplasmic DNA. CC: co-culture, MT: media treatment (media from the insert and the well given to naïve cells cultured in a separate plate). Exosome and EV DNA were amplified using an EGFP probe and probe-specific primers. EGFP-specific primers were used for the cytoplasmic DNA amplification. (**A**) CD63^+^ exosomes, (**B**) no immune separation after PEG-NaCl precipitation from the conditioned media, (**C**) cytoplasmic DNA after co-culture, and (**D**) cytoplasmic DNA after media treatment. Statistical analysis shows no significant difference between the Ct values of EGFP and EGFP-22mer transfected samples. A post hoc Dunnett’s test was used to compare the mean of the EGFP cassette-treated sample with the rest of the samples in the coculture experiments (**A**–**C**) as well as the media-treatment experiment (**D**). *p* value explanation is as follows: (**A**) Adjusted *p* value: *: *p* = 0.04, (**B**) Adjusted *p* value: *: *p* = 0.03, **: *p* = 0.002, (**C**) Adjusted *p* value: *: *p* = 0.02, **: *p* = 0.007, and (**D**) Adjusted *p* value: ns: *p* > 0.1. (ns = non-significant).

The qPCR analyses of the EVs and the exosomal DNA show no significance (ns) between the Ct values of the samples transfected with EGFP-22mer compared to the EGFP cassette alone, suggesting that the 22mer insert alone is not effective in DNA localization (Figure 8A,B). As expected, the Ct values of the cytoplasmic DNA shows no significance (ns). This is because the quantities of the EGFP-22mer DNA used in transfection are exactly the same (Figure 8C). The Ct values of the cytoplasmic DNA of the cells treated with the media (MT) from the insert or the well also show the same trait of no significance between the Ct values of EGFP-22mer and EGFP (Figure 8D).

### 2.7. Attachment of 22mer Insert Containing NANOGP8 3′-UTR Improves the Localization of a Non-Native EGFP DNA to EVs

**Figure 9:**
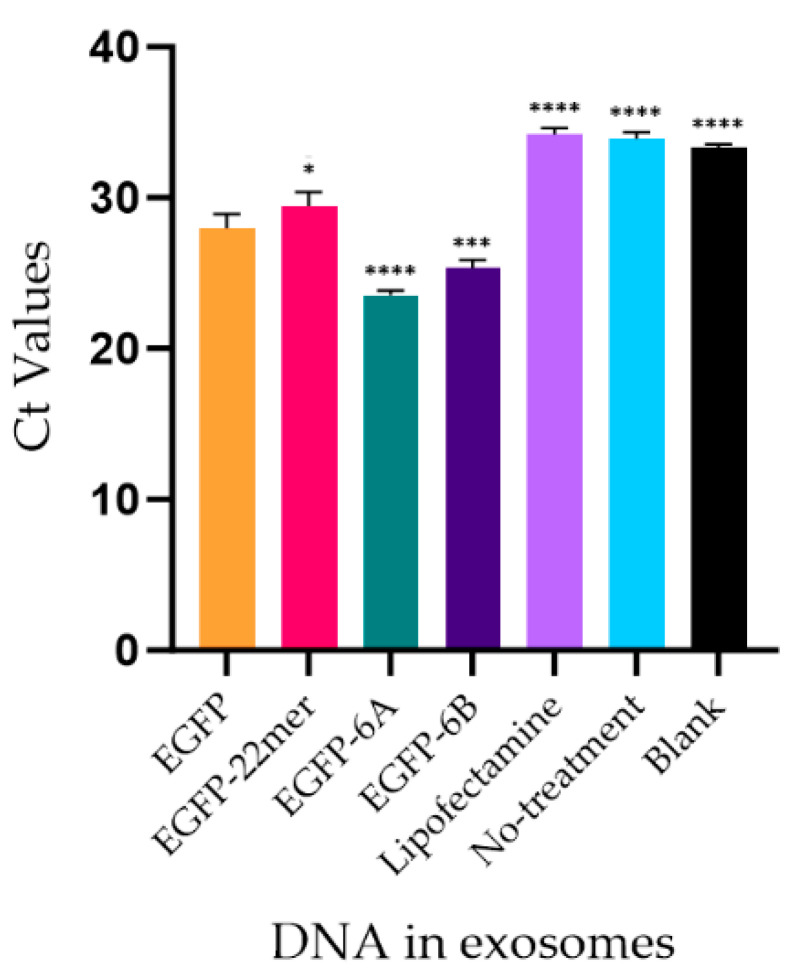
Graph showing the Ct values of exosomal EGFP DNA amplified via qPCR using an EGFP probe. Exosome samples used in the qPCR were normalized to 0.5 µg protein (A280) content. The significantly lower Ct value for the exosomal DNA of HEK cells transfected with EGFP-6A, where 6A is the NANOGP8 3′-UTR containing 22mer, indicates a higher amount of DNA targeted to the exosomes. This implies that the region with the 22mer has an exosome-localization capability. The graph also depicts that the 22mer by itself does not have an ability to sort the DNA to the exosomes. Ordinary One-way ANOVA was performed for multiple comparisons of other types of DNA to EGFP, followed by a post hoc Dunnett’s test to compare the mean of EGFP values to the rest of the DNA samples. ****: *p* < 0.0001, ***: *p* = 0.005, and *: *p* = 0.0386.

Although the 22mer insert targets more NANOG 3′-UTR DNA to the exosomes, the insert alone is not enough to act as a strong EV-localization sequence. Therefore, we wanted to confirm if the UTR flanking the 22mer has EV-localization properties. In order to check for the EV/exosome-localization efficacy of the 22mer and the flanking sequence, the PCR products of clones 6A (22mer present) and 6B (22mer absent) were attached to a non-native DNA, i.e., EGFP. We have shown that more EGFP DNA localizes to EVs when the UTR is attached. Nevertheless, the inclusion of 22mer further enhances the localization of the cassette DNA to the EVs. The EV samples were normalized to the protein content. The Ct values of the EGFP DNA are represented in the graph in Figure 9. It is noteworthy that in spite of the addition of the NANOG sequence, the EGFP gene cassette expresses the protein efficiently. 

## 3. Discussion

The delivery of a functional, promoter-driven DNA gene cassette to cells or tissues is essential in gene therapy [34,35]. Enhancing the efficacy of DNA molecule introduction to EVs could offer a promising drug delivery system. Therefore, identifying a nucleotide sequence capable of serving as an EV-localization signal for DNA is essential. In our previous research, we explored 248 base pair long fragments of the exosomal DNA flanking the 22mer, using the primers located at positions 1657-1905 [NANOGP8 mRNA (GenBank Accession # NM_001355281.1)] in GBM-secreted exosomes. We showed that the presence of a 22mer insert in the 3′-UTR of NANOGP8 made more NANOGP8 DNA localize to the exosomes [4]. We hypothesized that this 22mer possibly has an exosome-localization function. To test this hypothesis, we conducted a quantitative analysis of the exosomal DNA of the 3′-UTR in the current research (Figure 2B). The qPCR of the exosomal DNA showed no significant difference in Ct values of 6A and 6B samples when HEK293 cells were transfected with NANOGP8 3′-UTR containing 22mer (clone 6A) and without the 22mer (clone 6B). This data suggests that the presence of 22mer in the UTR does not increase the efficacy of its localization to the exosomes. However, even in the absence of 22mer, the 6B DNA added to the cytoplasm showed an ability to localize the endogenous NANOGP8 3′-UTR DNA to exosomes (Figure 2A). We checked the quantities of 22mer between the cytoplasm and the EVs after the transfection of the cells with the PCR products of 6A and 6B. With the addition of the 6B to the cells, the ratio of the Ct values for the 22mer DNA in EVs to the cell cytoplasm is the same as the innate 22mer, i.e., the Lipofectamine and the no-treatment samples (Figure 3). However, the addition of 22mer along with the UTR changes the ratio of the localization. The ratios of the Ct values of the NANOGP8 3′-UTR in the EV–cytoplasm indicate that the elevated concentration of the 22mer-containing UTR DNA in the cytoplasm does not proportionately increase its location to the EVs. Simply increasing the concentration of DNA in the cytoplasm may not be enough to make it localize to EVs.

To test the functionality of the NANOGP8 3′-UTR in localizing a non-native DNA to EVs, we attached the 270-base pair PCR product of clone 6A and the 248-base pair PCR product of clone 6B to the 3′ end of a reporter gene. In the functional assay, DNA of the EGFP reporter gene, fused with the NANOGP8 3’-UTR containing the 22-mer, exhibited higher efficacy in localizing the gene to EVs, compared to the gene sequence fused with the UTR without the 22-mer (Figure 9). The data also indicate that the 22mer alone attached to the EGFP gene cassette is not only insufficient for the DNA localization to EVs, but it in fact reduces the efficacy of the DNA localization. Where the addition of the NANOGP8 3′-UTR downstream of the EGFP gene cassette improves the gene’s localization to EVs, having a 22mer in the UTR further increases the localization efficacy significantly. Therefore, the flanking sequences of the NANOGP8-3′-UTR are essential for the 22mer to localize the DNA into EVs. It is quite possible that a larger, unexplored part of the exosomal NANOGP8 3′-UTR other than the 22mer is involved in the localization of DNA to EVs, including exosomes.

The EGFP gene cassette, driven by the CMV promoter (Figure 6) when endogenously packed by the EVs, was functional in the EV-recipient cells (Figure 5). The gene cassette delivered to the target cells using the exosomes was also active and transcription-ready (Figure 7). The low intensity of green fluorescence in the cells at 24 h of the exosome treatment was observed, and after the next few days, EGFP signals were increased as the exosome-delivered gene cassette was transcribed. The EGFP signal was strong and detectable in the cells even after 4 weeks. Although lacking direct evidence, the sustained expression of EGFP following DNA gene cassette delivery may be due to the continuous redistribution of the gene cassette due to its spontaneous encapsulation in the exosomes. This EGFP expression via the DNA cassette is significantly longer than the EGFP protein delivery, where the protein has a half-life of approximately 15–16 h [36]. This longer expression of the desired protein in the target cells could be one of the merits of the exosomal transfection of DNA.

The exogenous loading of DNA through methods such as electroporation, sonication, or liposome transfection has not only been demonstrated to be inefficient due to the lack of a targeted loading, but may also induce the aggregation of EVs, thereby diminishing their permeability to the cells and tissues [29,34]. Sutaria et al. have discussed the advantages of endogenous methods for RNA loading into EVs over exogenous methods [37]. Similarly, developing an endogenous method to load the DNA of interest into EVs would give a significant benefit to the fields of biotechnology and molecular medicine. Utilizing cellular machinery for the direct loading of nucleotides into the EVs without the need for post-secretion manipulation makes the process scalable as required and reduces the risk of contamination. Furthermore, the endogenous method to load DNA does not require the chemical modification of nucleotides or the EVs themselves, potentially mitigating unknown side effects, thus enhancing its clinical utility [29]. The EV delivery of DNA could also be safer than viral transduction since it may transiently affect downstream processes such as transcription and translation without the genomic integration [38,39]. 

## 4. Materials and Methods

### 4.1. Standard PCR Amplification of Exosomal NANOGP8 3′-UTR from pCR™4-TOPO™ TA Clones

In our previously published research, we directly amplified the exosomal DNA of cultured GBM cells using NANOGP8-3′-UTR-specific primers. The PCR product was cloned into the pCR™4-TOPO™ TA Vector and the clones were sequenced (4). Clone 6A, containing 22-base pair insert, (the insert referred to as “22mer”), and the clone 6B, without the 22mer, from the published research, were used as templates to reamplify the originally cloned PCR products (S3). The primers are as follows: Forward- 5’ GGATGGTCTCGATCTCCTGA 3’/reverse- 5’ CCCAATCCCAAACAATACGA 3’. Using GoTaq^®^ G2 Flexi DNA Polymerase (Promega, Cat. # M7808), the PCR reactions were set up for the thermocycling program: 94 °C for 5 min, (denaturation: 94 °C for 30 s, annealing: 55 °C for 30 s, Extension: 72 °C for 2 min) × 35, 72 °C for 10 min. The PCR products were electrophoresed on 1.5% Agarose gel in 1X TAE buffer. This DNA, and the rest of the DNA electrophoresed on the agarose gel in the research, are eluted from the gel using QIAquick PCR Purification Kit (QIAGEN, Cat. #28104), following the manufacturer’s protocol.

### 4.2. Creation of EGFP Gene Cassette

**Figure 10:**
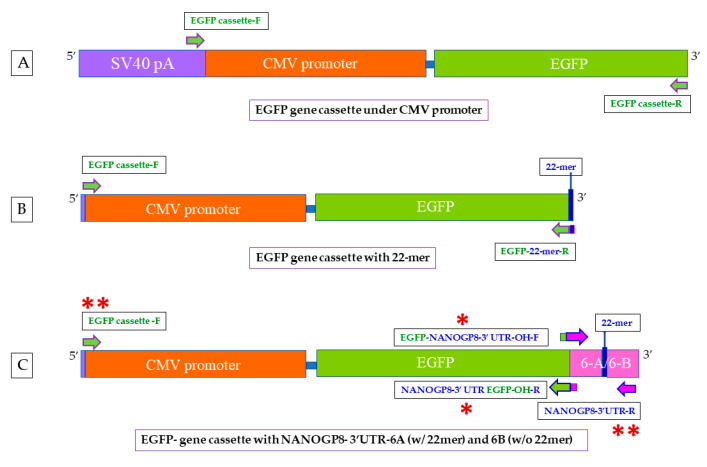
EGFP gene cassette design and the subsequent additions to the 3′ end. (**A**) EGFP gene cassette with the CMV promoter. The forward primer is located 47 base pairs upstream of the promoter, in SV40 late pA. (**B**) Addition of 22mer to the EGFP gene cassette: the 22mer itself was used as a primer. A short sequence of 3′ end of the EGFP cassette was included in the primer design. (**C**) The PCR products of clone 6A (with 22mer) and 6B (without 22mer) were fused with the EGFP gene cassette. The forward EGFP primer sits at the 3′ end of the cassette and contains an overhang (OH) of a short sequence of NANOGP8 3′-UTR. The reverse NANOGP8-3′-UTR primer is located at the 5′ end of the PCR product, with an OH of EGFP sequence. After the first PCR with the OH primers (one red asterisk), the fused product was reamplified with EGFP cassette forward primer (located in SV40 late pA) and the reverse primer for NANOGP8-3′-UTR (denoted with two red asterisks).

EGFP cassette expresses the gene under CMV promoter. Using Vector Builder’s pRP[Exp]-EGFP/Neo-CMV>{Tag/hNANOG[NM_024865.2]*} (referred to as XPAK-NANOGP8-EGFP in a shorter version) as template, we amplified the promoter and the downstream gene portion of the plasmid [4]. We created a forward primer located in the SV40 late pA, upstream of the CMV promoter (5′ GTGGGAGGTTTTTTAAAGCAAGTAA 3′). The reverse primer is located at the 3′ end of the EGFP gene (5′ CAGAGCAGCCGATTGTCTGT 3′) and excludes the Neomycin sequence. We did not reintroduce the stop codon that was eliminated with this exclusion (Figure 10A). The PCR reactions were set up as follows: 94 °C for 5 min, (denaturation: 94 °C for 30 s, annealing: 60 °C for 30 s, Extension: 72 °C for 2 min) × 35, 72 °C for 10 min. The PCR product was gel electrophoresed and eluted for downstream application.

### 4.3. Addition of 22mer and NANOGP8-3′-UTR (PCR Products of Clones 6A and 6B) to the EGFP Gene Cassette

*Adding 22mer to the EGFP cassette:* A forward primer sitting at the 5′ end of the CMV promoter (F: 5′ GTGGGAGGTTTTTTAAAGCAAGTAA 3′) and the 22mer sequence with an overhang of EGFP-3′ end (R: 5′ CTCTGGCTAAGGACAACATTGATAG 3′) were designed to attach the 22mer to the EGFP cassette (Figure 10B). Using the EGFP cassette as template, the cassette was PCR amplified as follows: 95 °C: 5 min, [95 °C: 30 s, 55 °C: 30 s, 72 °C: 2 min] × 35, 72 °C: 10 min. The PCR product was electrophoresed on a 1.5% agarose gel in 1X TAE buffer. It was eluted from the gel for transfections of the cells. 

*Adding 6A and 6B to the EGFP cassette:* The primers designed to contain overhangs of partial sequences of EGFP (3′ end) and NANOGP8 3′-UTR (5′ end) were used to fuse EGFP gene cassette with the PCR products of 6A and 6B clones (F: 5′ GGCTGCTCTGGGATGGTCTCGATCT 3′/R: 5′ AGATCGAGACCATCCCAGAGCAGCC 3′). The original CMV-EGFP gene cassette and the PCR products of 6A or 6B were added together as templates in the PCR mix. The following thermocycling program was used: 95 °C: 5 min, [95 °C: 30 s, 65 °C: 30 s, 72 °C: 90 s] × 10, 72 °C: 10 min. The PCR products were electrophoresed on 1.5% agarose gel in 1X TAE buffer. The QIAquick PCR Purification Kit (QIAGEN, Cat. # 28104) was used in eluting the DNA from the gel, following the manufacturer’s protocol (Figure 10C and Figure S4). The fusion product was re-amplified with the forward primer used in making the EGFP gene cassette (5′ GTGGGAGGTTTTTTAAAGCAAGTAA 3′) and the reverse primer used in exosomal NANOGP8 3′-UTR amplification of the clones 6A and 6B (R: 5’ CCCAATCCCAAACAATACGA 3’).

### 4.4. Cloning the EGFP-6A/6B into pCR™4-TOPO™ TA Vector for Sequencing

The re-amplified EGFP-6A/6B cassette, after elution from the agarose gel, was ligated with the pCR™4-TOPO™ TA sequencing vector following the manufacturer’s protocol (ThermoFisher Catalog number: 450030). Chemically competent E. coli strain Stbl3 cells were transformed with the ligations. The colonies were selected on 100 μg/mL Ampicillin containing LB agar plates with an overnight incubation at 37 °C. A colony PCR was performed to confirm the presence of the ligated product. The colonies (clones) were grown overnight at 37 °C in LB broth (100 μg/mL Ampicillin). The plasmid was extracted using a kit and the manufacturer’s protocol was followed (Qiagen Catalog number: 27104. QIAGEN GmbH, QIAGEN Strasse 1, 40724 Hilden, Germany). The clones were Sanger sequenced to confirm the presence of the PCR product (Figure S5). 

### 4.5. Restriction Enzyme (RE) Digestions of the pCR™4-TOPO™ TA-EGFP-6A/6B Clones

*EcoRI-digestion to release the cassette:* The pCR™4-TOPO™ TA sequencing vector contains two restriction enzyme (RE) digestion sites for enzyme EcoRI. The digestion with the enzyme releases the PCR product cloned into the vector. A total of 5 µg clone DNA was mixed with fast digest EcoRI enzyme and buffer (ThermoFisher Catalog number: FD0274). Following the incubation at 37 °C for 10 min, the samples were electrophoresed on 1.5% Agarose gel and eluted for transfecting the HEK293 cells (Figure S6). 

*HpyF3I-digestion for confirmation of the clones:* The sequencing vector, along with EGFP, has 9 cutting sites for HpyF3I. The 22mer in 6A has one RE site for the enzyme. A total of 1 µg DNA of each clone was digested with HpyF3I, using the same conditions as in EcoRI-digestions described above (Figure S7). 

### 4.6. HEK293 Cell Culture

HEK293 (ATCC catalog number: CRL-1573) cells were cultured in Dulbecco’s Modified Eagle Medium containing 10% FBS, 1% L-glutamine, 1% 100× nonessential amino acids, and 1% antibiotic/antimycotic. When the experiments involved EV collection or their further purification to exosomes, the exosome-depleted FBS was used. The cells were incubated in tissue culture-treated culture flasks and plates at 37 °C with 5% CO_2_. All the following experiments involving the HEK293 cell cultures use the same conditions. 

### 4.7. Testing the Functionality of the EGFP Gene Cassette

*Direct transfection with the cassette DNA using transfection reagent:* Using Lipofectamine™ 2000 Transfection Reagent (ThermoFisher catalog number: 11668019), and following the manufacturer’s protocol, HEK293 cells were transfected with the cassette DNA of EGFP, EGFP-22mer, EGFP-6A/6B, and the XPAK-NANOGP8-EGFP plasmid. The cells were imaged with a fluorescent microscope (Scope: Zeiss Observer.A1/camera: AxioCam ICc 3/LED Fluorescence light source: HXP 120 C), at a wavelength of Excitation 488 nm /Emission 509 nm. The cells were also exposed to the RFP laser line with Excitation 532 nm/Emission 588 nm. This was to confirm that the fluorescence from EGFP is not an artifact. 

### 4.8. Delivery via EVs in a Co-Culture or the Conditioned Media Treatment of the HEK293 Naïve Cells

**Figure 11:**
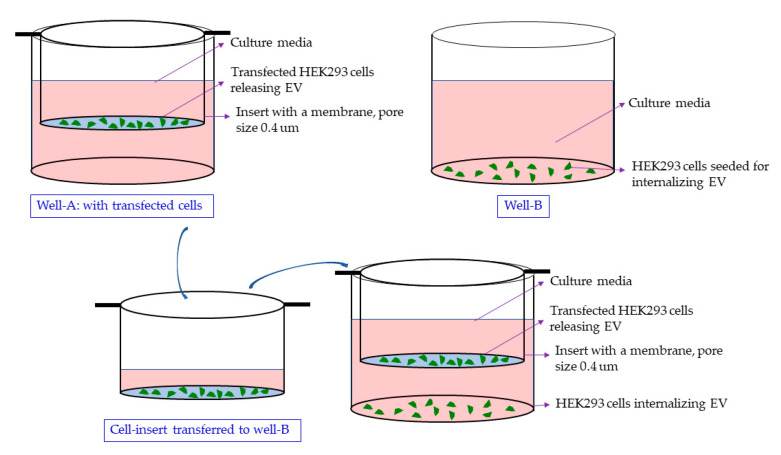
The co-culture setup. The HEK293 cells cultured in cell insert (pore size of 0.4 µm) are transfected separately and the insert is moved to the well containing naïve cells for co-culture. The cells in the insert and the well share the media allowing an exchange of EVs.

The HEK293 cells are seeded on the same day in the cell insert and the well. The cells in the cell insert are transfected in a separate plate to avoid an unintended exposure of the DNA to naïve cells in the well. Within 24 h, after visual confirmation of the EGFP expression, the insert was placed in the wells containing the naïve cells (Figure 11). The cells were co-cultured for 7 days. In the insert, a pore size of 0.4 µm prohibits the exchange of the cells. For the conditioned media treatment that does not involve a co-culture, the media was removed and centrifuged at 10,000× *g* for 30 min to remove the cell debris. Naïve HEK293 cells grown in a separate plate were treated with the supernatant. The cells were cultured in the conditioned media for five days.

### 4.9. Treatment of Naïve HEK293 Cells with EGFP Cassette Containing Exosomes Containing 

The HEK293 cells were cultured in a 96-well plate. At a cell confluency of 75%, 200 µL conditioned media was removed from the well. A 50 µL exosome suspension in 1× PBS was added to the well. The cells were incubated for 1 h and fresh 200 µL HEK media was added to the well followed by a continued incubation. The cells were imaged at 24 h, 72 h, and 5 days using fluorescence microscopy (Excitation 488 nm/Emission 509 nm). 

### 4.10. EV/Exosome Isolation

Using 20% Polyethylene Glycol (PEG8000) and 5M NaCl, EVs were precipitated from the conditioned media [40]. Using CD63 antibody conjugated magnetic beads (Exosome-Human CD63 Isolation/Detection Reagent from cell culture media, Thermo Scientific catalog number: 10606D), and following the manufacturer’s protocol, the exosomes were either directly separated from the conditioned media or further purified from the EV suspension. We have explained this process in details in a previous publication [41]. 

### 4.11. Standard PCR of Exosomal DNA Using CMV and EGFP Primers 

The HEK 293 cells were transfected with EGFP, EGFP-22mer cassettes, and the XPAK-NANOGP8-EGFP plasmid. Although, for the qualitative PCR, the CD63^+^ exosomes collected after transfections were normalized to their protein contents. Using the following primer pairs and the thermocycling conditions, the PCR amplification was performed. The amplification products were electrophoresed on agarose gel. Forward primer at CMV-3′ end (5′ GCGTGTACGGTGGGAGGTCT 3′)/reverse primer in the EGFP gene (5′ CTTGTACAGCTCGTCCAT 3′). Thermocycling: 95 °C: 5 min, [95 °C: 30 s, 52 °C: 30 s, 72 °C: 2 min] × 35 cycles, 72 °C: 10 min). Forward primer was located in the EGFP gene (5′ ATGGTGAGCAAGGGCGAG 3′)/reverse primer made of the 22mer sequence itself (5′ CTCTGGCTAAGGACAACATTGATAG 3′). Thermocycling: 5 min, [95 °C: 30 s, 55 °C: 30 s, 72 °C: 2 min] × 35 cycles, 72 °C: 10 min).

### 4.12. Standard PCR Amplification of Cytoplasmic DNA Using CMV and EGFP Primers 

The cytoplasmic DNA was extracted from the HEK293 cells after co-culture and media treatment using the plasmid DNA extraction kit (Qiagen Catalog number: 27104), following the manufacturer’s protocol. Forward primer at CMV-3′ end (5′ GCGTGTACGGTGGGAGGTCT 3′)/reverse primer in the EGFP gene (5′ CTTGTACAGCTCGTCCAT 3′). Thermocycling: 95 °C: 5 min, [95 °C: 30 s, 52 °C: 30 s, 72 °C: 2 min] × 35 cycles, 72 °C: 10 min).

### 4.13. qPCR of EGFP DNA in EV/Exosomes Using EGFP Probe

The EV/exosomes were normalized to their protein content. Using the forward primer 5′ AGTCCGCCCTGAGCAAAGA 3′, the reverse primer 5′ TCCAGCAGGACCATGTGATC 3′, and the probe CCCAACGAGAAGCG, the EGFP DNA was amplified in QuantStudio 7 thermocycler. The thermocycling conditions were as follows: 95 °C: 2 min, 95 °C: 15 s, 60 °C: 30 s.

Reaction mix: AzuraQuant Probe 1-Step qPCR Mix (Azura catalog number: AZ4101). RTase enzyme was not used since the DNA was amplified directly.

### 4.14. qPCR for the Quantification of NANOGP8 UTR in EV/Exosomes upon Transfection with the PCR Products of Clones 6A and 6B 

The EVs collected from the conditioned media were further purified using CD63 antibody for immune-capture and directly used in the qPCR without the DNA isolation.

The HEK293 cells transfected with the PCR products of exosomal NANOGP8 3′-UTR DNA clones 6A (22mer present) and 6B (22mer absent) secreted exosomes were normalized to protein content and used directly for qPCR amplification, without isolating the DNA. The NANOGP8 3′-UTR specific primers used in the amplification are from the published research (Forward: 5’ GGATGGTCTCGATCTCCTGA 3′/reverse: 5’ CCCAATCCCAAACAATACGA 3’) (Figure 2B). The reverse primer in Figure 2A is the 22mer itself (5′ GCTTCTATCAATGTTGTCCTTAGC 3′). The AzuraQuant™ Green Fast qPCR Mix LoRox (Azura catalog number: AZ-2101) was used for the PCR.

### 4.15. qPCRs Quantification of Cytoplasmic DNA

Nine days after transfection, i.e., seven days of co-culture and five days of media treatment, the cytoplasmic DNA was extracted using the plasmid DNA extraction kit (Qiagen Catalog number: 27104) following the manufacturer’s protocol. A total of 25 ng of cytoplasmic DNA of co-cultured cells and the conditioned media-treated cells were amplified using GFP primers (Forward: 5′ ATGGTGAGCAAGGGCGAG 3′/reverse: 5′ CTTGTACAGCTCGTCCAT 3′). In QuantStudio 7 thermocycler, the conditions were set as follows: 95 °C: 5 min, [95 °C: 15 s, 60 °C: 30 s, 72 °C: 30 s] × 35 cycles).

### 4.16. qPCRs of Cytoplasmic and EV DNA for 22mer Quantification and Comparison 

A total of 2 days after transfection of the HEK293 cells with the PCR products of 6A and 6B clones, cytoplasmic DNA was extracted following the same protocol as in Section 4.15. The EVs were precipitated using the PEG/NaCl method, described previously in Section 4.10. The EVs were normalized to their protein content and used directly to access the DNA cargo as template without DNA extraction. Just as in previous qPCRs, AzuraQuant™ Green Fast qPCR Mix LoRox (Azura catalog number: AZ-2101) was used. The forward primer was from NANOG 3′-UTR and the 22mer itself was employed as the reverse primer. The primer sequences are as follows: Forward: 5′ GGATGGTCTCGATCTCCTGA 3′, and reverse- 5′ GCTTCTATCAATGTTGTCCTTAGC 3′. Thermocycling conditions in the QuantStudio 7 thermocycler were set as follows: 95 °C: 10 min, [95 °C: 15 s, 64 °C: 30 s] × 40 cycles).

## 5. Conclusions

In summary, our research unveils the remarkable potential of the NANOGP8 3′-UTR-22mer sequence when fused with DNA fragments, showcasing its ability to enrich DNA, specifically within EVs. This groundbreaking approach promises precise DNA delivery to target cells, making it a game-changer for genetic engineering and DNA-based drug delivery strategies. By tapping into the natural transport capabilities of EVs, this pioneering DNA localization technique paves the way for innovative therapeutic interventions. It enables the efficient and targeted transfer of genetic material to cells of interest, holding transformative potential for fields as diverse as biomedicine and regenerative medicine.

As we push the boundaries of this technology, the scope of its application continues to expand, heralding a new era of medical advancements. Moreover, the non-invasive nature of this EV-mediated DNA delivery, which eschews the need for chemical modifications, significantly reduces the risk of side effects, thereby enhancing the safety profile of these cutting-edge therapeutic approaches.

## Data Availability

The data presented in this study are available in the article. The original law data are available on request from the corresponding author.

## Supplementary Materials

The following supporting information can be downloaded at: https://www.mdpi.com/article/10.3390/ijms25137294/s1.
