# Supplementary material for: 3′-UTR Sequence of Exosomal NANOGP8 DNA as an Extracellular Vesicle-Localization Signal
Source: Int J Mol Sci. 2024 Jul 2;25(13):7294. doi: 10.3390/ijms25137294 (PMC11242200; doi:10.3390/ijms25137294)
Supplement: Supplementary file 1 [file ijms-25-07294-s001.zip › S6.pdf]

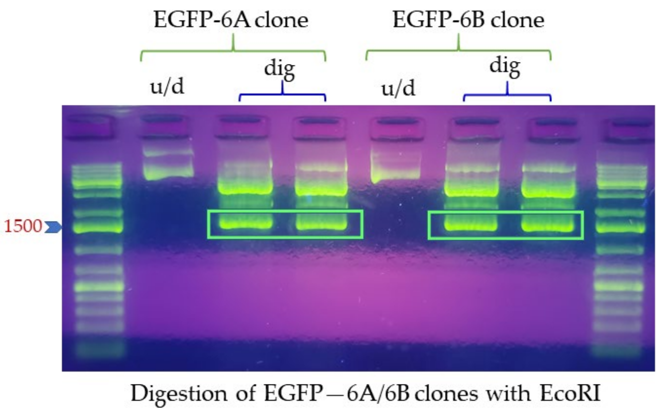

Fig S6. Digestion of the EGFP-6A/6B-pCR™4-TOPO™ TA clones with restriction enzyme EcoRI to release the cassette. The DNA bands in green frames were eluted for transfection of HEK293 cells.
