# Supplementary material for: 3′-UTR Sequence of Exosomal NANOGP8 DNA as an Extracellular Vesicle-Localization Signal
Source: Int J Mol Sci. 2024 Jul 2;25(13):7294. doi: 10.3390/ijms25137294 (PMC11242200; doi:10.3390/ijms25137294)
Supplement: Supplementary file 1 [file ijms-25-07294-s001.zip › S7.pdf]

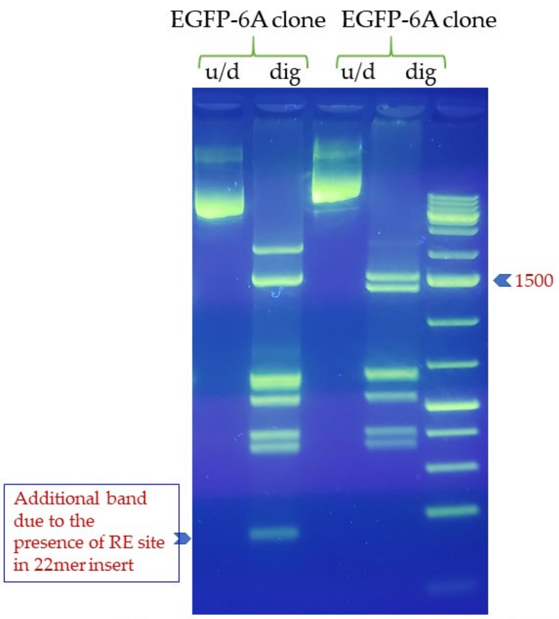

Digestion of EGFP—6A/6B clones with HpyF3I

Fig S7. HpyF3I-digestion for confirmation of the pCR™4-TOPO™ TA-EGFP-6A/6B clones: The sequencing vector, along with EGFP has 9 cutting sites for HpyF3I. the 22mer in 6A has one RE site for the enzyme and shows an additional band after digestion.
