# Supplementary material for: 3′-UTR Sequence of Exosomal NANOGP8 DNA as an Extracellular Vesicle-Localization Signal
Source: Int J Mol Sci. 2024 Jul 2;25(13):7294. doi: 10.3390/ijms25137294 (PMC11242200; doi:10.3390/ijms25137294)
Supplement: Supplementary file 1 [file ijms-25-07294-s001.zip › S1.pdf]

CMV-EGFP-cassette (sequenced with Reverse primer: EGFP-cassette-R), the sequence sent by GENEWIZ® from Azenta Life Sciences:

>EGFP-cassette-4-mine_H10.ab1

NNNNNNNNNNNAANNGCCNNNNNCNCCAAGCGGCCGGAGAACCTGCGTGCANNCCATTCTTTGTTCAATCATCTTGT
ACAGCTCGTCCATGCCGAGAGTGATCCCGGCGGCGGTCACGAACTCCAGCAGGACCATGTGATCGCGCTTCTCGTTG
GGGTCTTTGCTCAGGGCGGACTGGGTGCTCAGGTAGTGGTTGTCGGGCAGCAGCACGGGGCCGTCGCCGATGGGGGT
GTTCTGCTGGTAGTGGTCGGCGAGCTGCACGCTGCCGTCCTCGATGTTGTGGCGGATCTTGAAGTTCACCTTGATGC
CGTTCTTCTGCTTGTCGGCCATGATATAGACGTTGTGGCTGTTGTAGTTGTACTCCAGCTTGTGCCCCAGGATGTTG
CCGTCCTCCTTGAAGTCGATGCCCTTCAGCTCGATGCGGTTCACCAGGGTGTCGCCCTCGAACTTCACCTCGGCGCG
GGTCTTGTAGTTGCCGTCGTCCTTGAAGAAGATGGTGCGCTCCTGGACGTAGCCTTCGGGCATGGCGGACTTGAAGA
AGTCGTGCTGCTTCATGTGGTCGGGGTAGCGGCTGAAGCACTGCACGCCGTAGGTCAGGGTGGTCACGAGGGTGGGC
CAGGGCACGGGCAGCTTGCCGGTGGTGCAGATGAACTTCAGGGTCAGCTTGCCGTAGGTGGCATCGCCCTCGCCCTC
GCCGGACACGCTGAACTTGTGGCCGTTTACGTCGCCGTCCAGCTCGACCAGGATGGGCACCACCCCGGTGAACAGCT
CCTCGCCCTTGCTCACCATGGTGGCGCAGTGGGTTCTCTAGTTAGCCAGAGAGCTCTGCTTATATAGACCTCCCACC
GTACACGCCTACCGCCCATTTGCGTCAATGGGGGCGGAGTTGTTACGACATTTTGGAAAGTCCCGTTGATTTTGGTG
CCAAAACAAACTCCCATTGACGTCAATGGGGTGGANACTTNGNAATCCCCGTGAGTCAAACCGCTATCCACGCNCAT
TGATGTACTGCCAAAACCGCATCACCATGGNAATANCNATGACTN

(A)

Standard BLAST

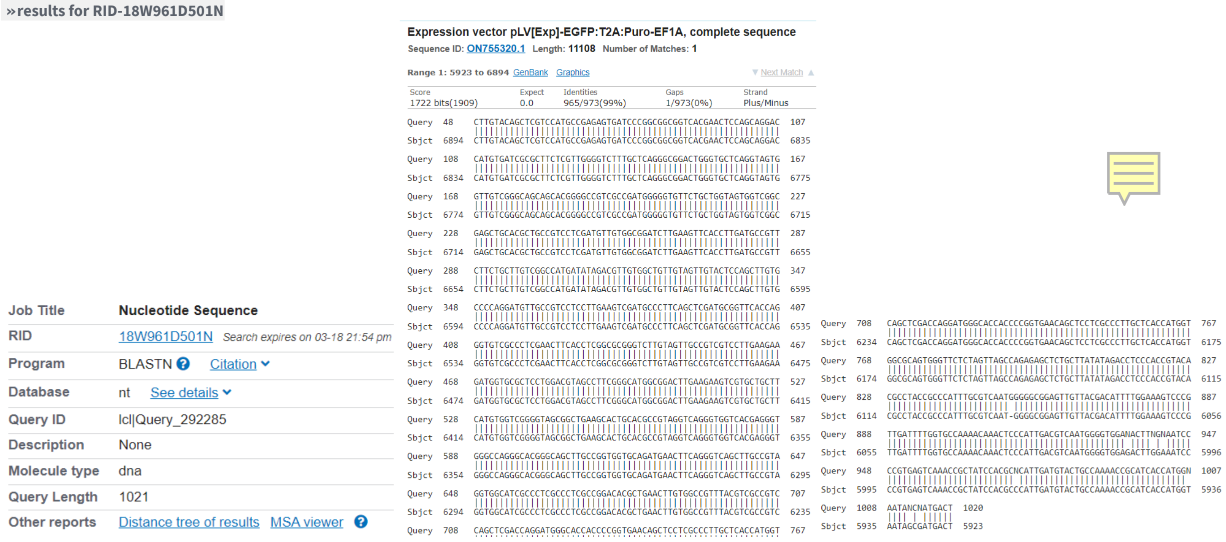

(B)

Standard BLAST comparison with the template plasmid pRP[Exp]-EGFP/Neo-CMV>{Tag/hNANOG[NM_024865.2]*} :

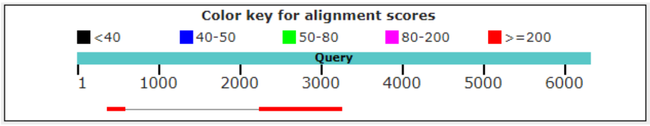

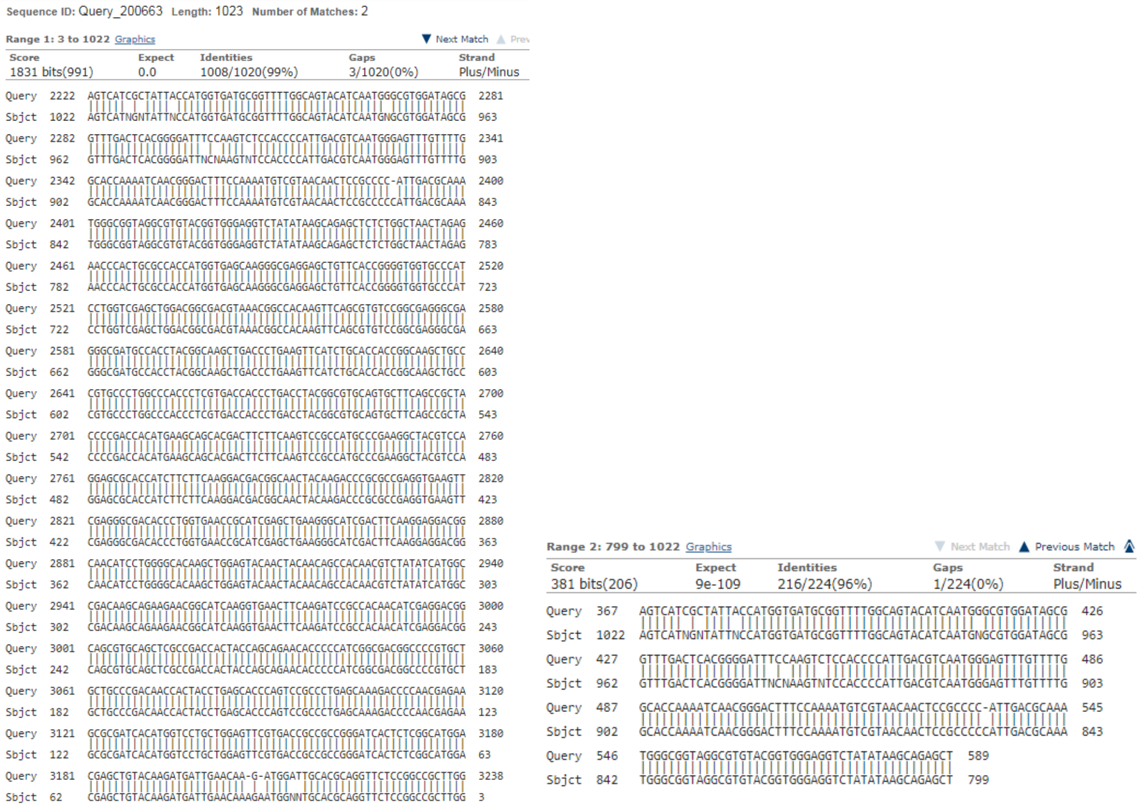

Fig S1: (A) Standard and (B) comparative BLAST of Sanger sequenced PCR product obtained using Vector Builder's pRP[Exp]-EGFP/Neo-CMV>{Tag/hNANOG[NM_024865.2]* plasmid (referred to as XPAK-NANOGP8-EGFP in the manuscript), (vector ID # VB170211-1008gnb) as template.
