# Supplementary material for: 3′-UTR Sequence of Exosomal NANOGP8 DNA as an Extracellular Vesicle-Localization Signal
Source: Int J Mol Sci. 2024 Jul 2;25(13):7294. doi: 10.3390/ijms25137294 (PMC11242200; doi:10.3390/ijms25137294)
Supplement: Supplementary file 1 [file ijms-25-07294-s001.zip › S2.pdf]

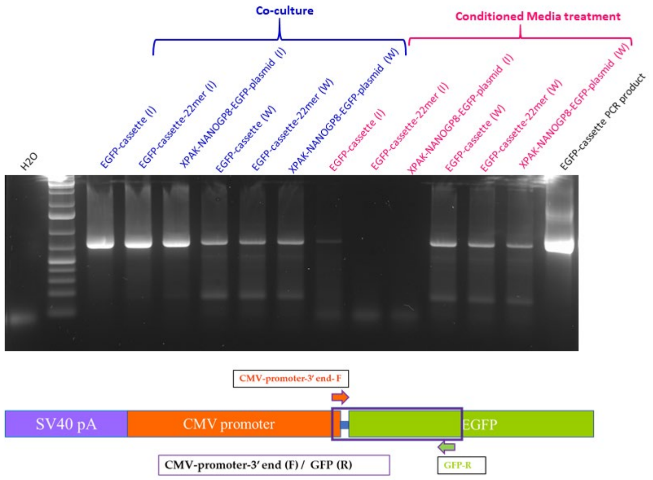

Fig S2. A standard PCR of miniprepped, EGFP cassette cytoplasmic DNA of HEK293. Upon coculture of EGFP cassette-transfected and the naïve cells, the conditioned media from the insert and the well were given to a separate set of HEK293 naïve cells, labelled as conditioned media treatment. The cytoplasmic DNA of the cells was collected using a DNA plasmid miniprep kit. The cytoplasmic DNA was PCR amplified using a primer set, where the forward primer is located in the 3′ end of the CMV promoter. The reverse primer sits in the EGFP gene.
