# Supplementary material for: 3′-UTR Sequence of Exosomal NANOGP8 DNA as an Extracellular Vesicle-Localization Signal
Source: Int J Mol Sci. 2024 Jul 2;25(13):7294. doi: 10.3390/ijms25137294 (PMC11242200; doi:10.3390/ijms25137294)
Supplement: Supplementary file 1 [file ijms-25-07294-s001.zip › S3.pdf]

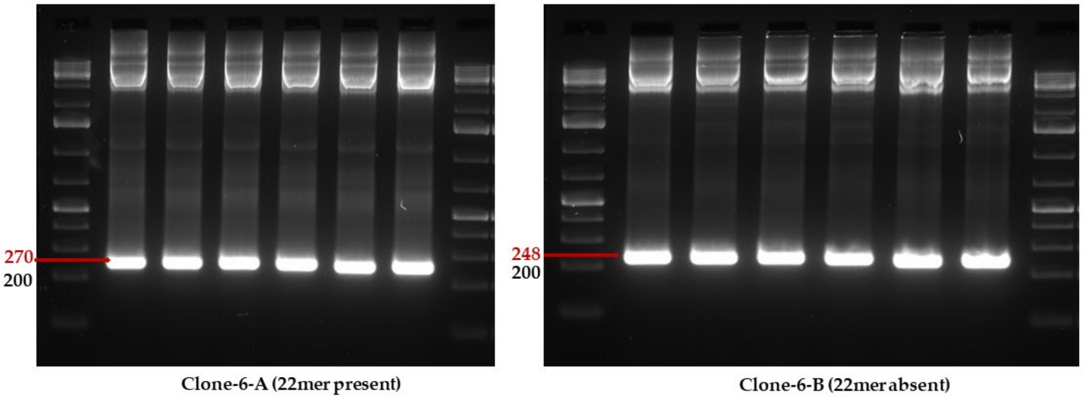

Fig S3. Standard PCR amplification of pCR4-TOPO-TA clones of exosomal NANOGP8 3' UTR of Glioblastoma multiforme cells. Clone 6-A, containing 22-base pair insert, (the insert referred to as simply a "22mer"), and the clone 6-B, without the 22mer, from the published research were used as templates to reamplify these originally cloned PCR products.
