# Supplementary material for: 3′-UTR Sequence of Exosomal NANOGP8 DNA as an Extracellular Vesicle-Localization Signal
Source: Int J Mol Sci. 2024 Jul 2;25(13):7294. doi: 10.3390/ijms25137294 (PMC11242200; doi:10.3390/ijms25137294)
Supplement: Supplementary file 1 [file ijms-25-07294-s001.zip › S4.pdf]

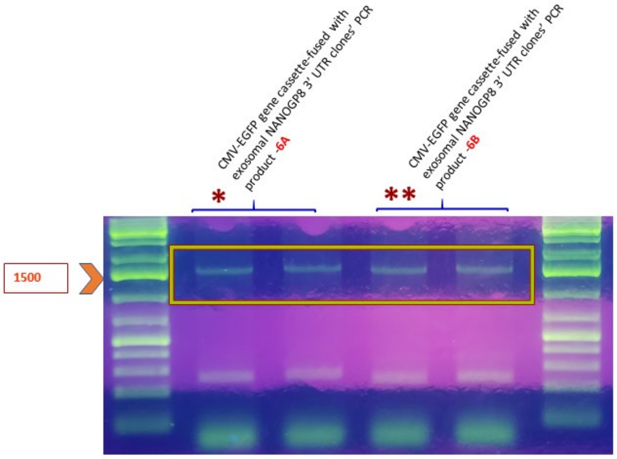

Fig S4. Adding the PCR product of the exosomal NANOGP8 3′ UTR clones 6A and 6B to the EGFP cassette: The primers designed to contain overhangs of partial sequences of EGFP (3′ end) and NANOGP8 3′UTR (5′ end) were used to fuse EGFP gene cassette with the PCR products of 6-A and 6-B clones.
