# Supplementary material for: 3′-UTR Sequence of Exosomal NANOGP8 DNA as an Extracellular Vesicle-Localization Signal
Source: Int J Mol Sci. 2024 Jul 2;25(13):7294. doi: 10.3390/ijms25137294 (PMC11242200; doi:10.3390/ijms25137294)
Supplement: Supplementary file 1 [file ijms-25-07294-s001.zip › S5.pdf]

>EGFP-6A-clone-8-M13-R-M13R_D05.ab1

NNNNNNNNNNNNNTANCCTCNCTAAAGGGACTAGTCCTGCAGGTTTAAACGAATTCGCCCTTGGATGGTC
TCGATCTCCTGACCTTGTGATCCGCCCGCCTCGGCCTCCCTAACAGCTGGGATTACAGGCGTGAGCCACC
GCGCCCTGCCTAGAAAAGACATTTTAATAACCTTGGCTGCTAAGGACAACATTGATAGAAGCCGTCTCTG
GCTATAGATAAGTAGATCTAATACTAGTTTGGATATCTTTAGGGTTTAGAATCTAACCTCAAGAATAAGA
AATACAAGTACGAATTGGTGATGAAGATGTATTCGTATTGTTTGGGATTGGGAAGGGCGAATTCGCGGCC
GCTAAATTCAATTCGCCCTATAGTGAGTCGTATTACAATTCACTGGCCGTCGTTTTACAACGTCGTGACT
GGGAAAACCCTGGCGTTACCCAACTTAATCGCCTTGCAGCACATCCCCCTTTCGCCAGCTGGCGTAATAG
CGAAGAGGCCCGCACCGATCGCCCTTCCCAACAGTTGCGCAGCCTATACGTACGGCAGTTTAAGGTTTAC
ACCTATAAAAGAGAGAGCCGTTATCGTCTGTTTGTGGATGTACAGAGTGATATTATTGACACGCCGGGGC
GACGGATGGTGATCCCCCTGGCCAGTGCACGTCTGCTGTCAGATAAAGTCTCCCGTGAACTTTACCCGGT
GGTGCATATCGGGGATGAAAGCTGGCGCATGATGACCACCGATATGGCCAGTGTGCCGGTCTCCGTTATC
GGGGAAGAAGTGGCTGATCTCAGCCACCGCGAAAATGACATCAAAAACGCCATTAACCTGATGTTCTGGG
GAATATAAATGTCNNNATGAGATTATCAAAAAGGATCTTCNCCTAGATCCTTTTCACGTAGAAAGCCAGT
CCGCANAAANNTGCTGACCCCGGATGAATGTCAGCTACTGGGCTATCTGGANNANGNAAACNCAAGCNCA
AANNNAANCNNNAGCTTGCAGTNGGNTTACATGGCGATAGCTAN

**(A)** Comparative BLAST between EGFP cassette sequence and the EGFP-NANOGP8 3′ UTR clones:

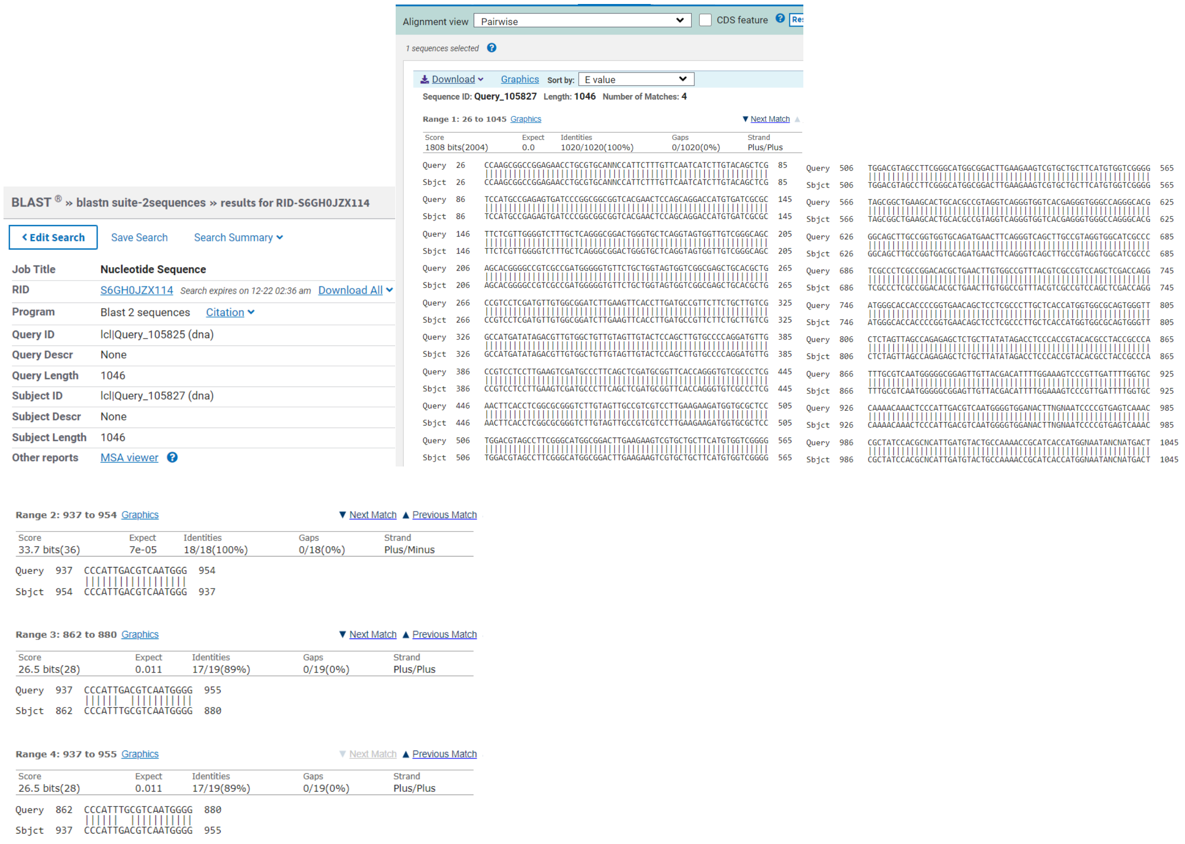

(B) Comparative BLAST between NANOGP8 3′ UTR sequence and the EGFP-NANOGP8 3′ UTR clones. The highlighted region denotes the 22mer insert:

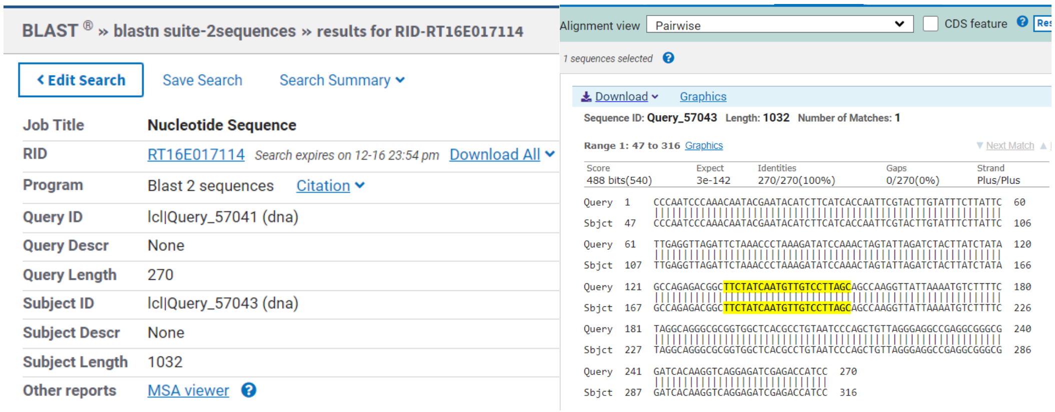


**Fig S5. A typical BLAST analysis of the pCR™4-TOPO™ TA Vector-EGFP-6A/6B cassette clones.** The re-amplified EGFP-6A/6B cassette was cloned into the pCR4TOPO-TA sequencing vector and the clones were Sanger sequenced using universal M13 primers to confirm the presence of the PCR product.
